# Thyroid Feeding in Skin Diseases

**Published:** 1894-01-27

**Authors:** 


### THYROID FEEDING IN SKIN DISEASES.

It will, we think, be useful to follow up our article on the thyroid treatment of myxœdema (No. 372, vol. xv. November 11th, 1893) by some remarks on the application of this treatment to skin diseases. It must be understood, however, that this is still on its trial, and the cases recorded have been as yet insufficient to warrant such a belief in its applicability to cutaneous scaly disorders as to myxœdema.

The effect of administering thyroid extract in any form, whether by mouth or subcutaneously, is at once very marked on the skin. The skin becomes warmer and the sweat glands secrete copiously, and this effect is produced in healthy persons as well as in the myxœdematous.

Dr. Byrom Bramwell, of Edinburgh, having noticed this marked skin reaction, and that if the treatment was pushed or continued for some time, profuse desquamation followed, was led to attempt its use on various skin affections, a fine piece of deductive reasoning. He treated three cases of psoriasis of exceptional severity by raw thyroid feeding, two of them with most excellent results; the third seemed rather worse than better for the treatment.

From the effects invariably produced on the skin, it seemed natural to try thyroid ingestion in any skin diseases characterised by a scaly, dry, thickened epidermis, with a weak, feebly-nourished dermis beneath, such as psoriasis, xeroderma, or ichthyosis, and chronic eczema. Quite recently Dr. Leslie Phillips, of Birmingham, has published a series of seven cases (five being psoriasis, one xeroderma, and one chronic eczema), in all of which the thyroid treatment was tried, but only the xeroderma was benefited. By xeroderma we must be understood to mean* that dry, atrophic condition of the skin, always congenital, which is a mild degree of ichthyosis; it is a very common malady, and even in slight degrees, or only in a patchy form, is quite capable of producing great discomfort or annoyance. Moreover, the usual treatment, though capable in all cases of giving great relief, when properly and systematically carried out, is irksome and takes much time. This treatment involves frequent bran or alkaline baths, followed by careful inunction with glycerine, oil (almond, olive, or, better still, liquid vaseline), or (as recommended by Kaposi) an ointment containing 5 per cent. of naphthol. The condition is so inveterate that immediate relapse follows any neglect of these applications, and no internal remedies are of the least use; we must, therefore, hail with pleasure any simplification of treatment in these troublesome cases. It is unlikely that the good result from thyroid feeding will be more permanent than that produced by external emollients, because there is no doubt in these cases a congenital maldevelopment of the sweat glands; but it is not theoretically impossible that continual stimulation of the sweat glands by the administration of thyroid extract may, in time, produce a permanent increase in their secreting power. At the same time, as long as we know so little about the physiological properties of this extract, and what effect it may have on the nervous system generally, we must warn our readers that it is not a remedy to be lightly or carelessly administered, and should certainly never be employed unless under the careful and frequent observation of a capable physician. There is already a certain amount of evidence that the exhibition of thyroid gland in too large doses, or for too long a time, may produce symptoms resembling exactly the leading features of Graves's disease, or exophthalmic goitre. This was stated on the high authority of Dr. Murray, of Newcastle, in a discussion last month at the London Medical Society on Mr. Maude's paper upon "Exophthalmic Goitre," and confirmed by Mr. Maude.

The writer of this article has seen very dangerous symptoms resulting from the administration of overdoses of thyroid, given by the stomach. In one case, after two whole thyroids, the pulse ran up to 150, and great prostration and collapse ensued. We must also caution readers against using thyroids, whether whole or compressed, which are not absolutely fresh; if at all suspicious they may set up very troublesome diarrhœa.

In psoriasis, the effect of thyroid feeding has varied much, but it has been marvellously successful in several most severe and obstinate cases, which had resisted all other treatment. Here, again, we are glad to hail any new remedy, as psoriasis is so chronic, and often so disfiguring a skin disease, while the proper local treatment is sometimes so tedious; moreover, some of the most reliable external applications, such as tar ointments, or chrysophannic acid (the old Indian remedy, "goa-powder"), require very careful use lest the skin should be over-irritated, and they also stain and soil both skin and linen.

*For the very rare condition, properly known as "Atrophoderma Pigmentosum," is sometimes called xeroderma.

